# Cost Effective Synthesis of Graphene Nanomaterials for Non-Enzymatic Electrochemical Sensors for Glucose: A Comprehensive Review

**DOI:** 10.3390/s22010355

**Published:** 2022-01-04

**Authors:** Georgia Balkourani, Theodoros Damartzis, Angeliki Brouzgou, Panagiotis Tsiakaras

**Affiliations:** 1 Laboratory of Alternative Energy Conversion Systems, Department of Mechanical Engineering, University of Thessaly, Pedion Areos, 38334 Volos, Greece; gbalkourani@gmail.com; 2 Industrial Processes and Energy Systems Engineering, Institute of Mechanical Engineering, Sion, Ecole Polytechnique Fédérale de Lausanne, 1015 Lausanne, Switzerland; theodoros.damartzis@epfl.ch; 3 Department of Energy Systems, School of Technology, University of Thessaly, Geopolis, Regional Road Trikala-Larisa, 41500 Larisa, Greece; 4 Laboratory of Materials and Devices for Electrochemical Power Engineering, Institute of Chemical Engineering, Ural Federal University, 19 Mira Str., 620002 Yekaterinburg, Russia; 5 Laboratory of Electrochemical Devices Based on Solid Oxide Proton Electrolytes, Institute of High Temperature Electrochemistry (RAS), 620990 Yekaterinburg, Russia

**Keywords:** graphene-based nanomaterials, synthesis, reduced graphene oxide, glucose electrooxidation mechanism, electrochemical sensor, cobalt oxide nanomaterial, copper oxide nanomaterial, nickel oxide nanomaterial, direct growth, in-situ growth, laser-induced, polymer functionalized

## Abstract

The high conductivity of graphene material (or its derivatives) and its very large surface area enhance the direct electron transfer, improving non-enzymatic electrochemical sensors sensitivity and its other characteristics. The offered large pores facilitate analyte transport enabling glucose detection even at very low concentration values. In the current review paper we classified the enzymeless graphene-based glucose electrocatalysts’ synthesis methods that have been followed into the last few years into four main categories: (i) direct growth of graphene (or oxides) on metallic substrates, (ii) in-situ growth of metallic nanoparticles into graphene (or oxides) matrix, (iii) laser-induced graphene electrodes and (iv) polymer functionalized graphene (or oxides) electrodes. The increment of the specific surface area and the high degree reduction of the electrode internal resistance were recognized as their common targets. Analyzing glucose electrooxidation mechanism over Cu- Co- and Ni-(oxide)/graphene (or derivative) electrocatalysts, we deduced that glucose electrochemical sensing properties, such as sensitivity, detection limit and linear detection limit, totally depend on the route of the mass and charge transport between metal(II)/metal(III); and so both (specific area and internal resistance) should have the optimum values.

## 1. Introduction

Glucose is a complex molecule, containing six carbon atoms, which is difficult to split, via controlled stepwise oxidation. Consequently, facilitating the glucose electrooxidation reaction (GER) and the further evolution of the enzymeless glucose sensors, remains a challenge to the scientists. The main key requirements for the development of any high performance enzymeless electrochemical sensor are [1,2,3,4]: (1) the availability of as many as possible number of active sites onto the electrocatalytic surface area for the adsorption and diffusion of as many as possible reactant molecules, (2) the high electrocatalytic active sites’ capacity for the evolution of a multi-step electrooxidation reaction and (3) the fast charge transfer.

In the last years, according to literature research works [5,6], the activity of non-precious metals (and their oxides) towards GER seem to be greatly enhanced when they are combined with a graphene (or its derivatives)-based support material [7]. Namely, cobalt, nickel and copper (or their oxides) have attracted much more interest [8] since when they are supported on a different than graphene (or graphene oxide) material, their activity towards GER is very low, but when graphene material is engaged those non-precious metals present radically superior performance as electrochemical sensors. In particular, the high conductivity of graphene material in combination with its very large surface area increase the number of electron transfer as well as the amount of mass transport, improving glucose electrochemical sensors sensitivity, response time and stability. Moreover, due to its large pores, and the facilitated greater analyte transport the detection ability of graphene-based electrodes increases, reaching very low limit of detection, even in the range of pM [9]. Additionally, non-precious metals present lower chemisorption ability of various interfering substances, such as other molecules, proteins, etc., included into the human fluids [10], and so they constitute better candidates as electrodes for glucose electrochemical sensors. Besides the above-mentioned improved characteristics that a graphene-based electrode presents, the inclusion of non-precious metals lowers the total cost, making this approach (development of non-precious graphene-based electrodes) even more attractive.

Therefore, the perspectives of non-precious graphene (or oxides)-based electrodes concerning their application into enzymeless electrochemical glucose sensors ‘field, are huge. Because of this fact many research groups have focused on the amelioration of such electrode’s characteristics, proposing new synthesis route methods. In the current review paper, we mainly focused on the last three years works and we tracked mainly four different synthesis methods that have been explored for the development of non-precious graphene-based glucose sensing electrodes: (i) direct growth of graphene (or oxides) on metallic substrates, (ii) in-situ growth of metallic nanoparticles into graphene (or oxides) matrix, (iii) laser-induced graphene electrodes and (iv) polymer functionalized graphene (or oxides) electrodes. Additionally, we discuss glucose electrooxidation mechanism over Co-, Ni-, Cu- (and their oxides) graphene (or its oxides)-based electrocatalysts. Finally, the status and future perspectives of the non-precious graphene (or oxides)-based glucose sensing electrodes, are outlined.

### 1.1. Glucose Electrooxidation Mechanism over Co-Oxides/Graphene (or Its Derivatives)-Based Nanomaterials

Graphene (or its derivatives) support is the element of the metal (or oxide)/graphene-based electrode that mainly contributes to quick charge transfer during analyte molecule sensing process [11]. In addition, as known [12] metal (or oxides) electro-catalyze the reaction of glucose electrooxidation, therefore Co-oxides nanoparticles and its synergy with graphene material constitutes one of the most studied elements along with the Ni and Cu ones. However, GER mechanism over Co (or oxide)/graphene (or its derivatives)-based electrocatalysts is still under investigation. In literature, Co_3_O_4_ and Co(OH)_2_ [13,14,15,16] are the most common studied electrode materials onto various supports; since those are very effective towards GER and can be prepared with several techniques. 

Over the Co_3_O_4_-based electrodes in 0.1 M NaOH environment (pH ≈ 13), the majority of the research groups report a three-step glucose sensing mechanism [17]:

(1) $${\mathrm{Co}}_{3}O_{4}{+\mathrm{OH}}^{-}{+H}_{2}O\to {3\mathrm{CoOOH}+e}^{-}$$

(2) $${\mathrm{CoOOH}+\mathrm{OH}}^{-}\to {\mathrm{CoO}}_{2}{+H}_{2}{O+e}^{-}$$

(3) $${2\mathrm{CoO}}_{2}{+C}_{6}H_{12}O_{6}\to {2\mathrm{CoOOH}+C}_{6}H_{10}O_{6}$$

Under alkaline environment, the higher oxidation states of the metallic sites are necessary in order the GER to be promoted. According to the described GER mechanism (Equations (1)–(3)), the formation of CoO_2_/C_6_H_12_O_6_ complex plays a critical role. Moreover, as it is observed the GER mechanism is a multi-step electron transferring process requiring a lot of active sites as well as abundant electroactive surface area. This fact maybe explains the improved performance of the non-precious electrodes when supported onto graphene (or its derivatives) material. 

Specifically, reduced graphene oxide (rGO) is reported [18,19] as an excellent support for NPMs (such as Co, Ni, Cu, etc.), developing a synergistic mechanism with the metallic nanoparticles, that prevents the metallic nanoparticles from forming agglomerations when being deposited onto it, while facilitating quick electron transfer further forwarding GER. 

According to other research groups [19] GER mechanism over Co_3_O_4_ onto rGO and onto GO, is described by the following equations:

(4) $${\mathrm{CoOOH}+\mathrm{OH}}^{-}\to {\mathrm{CoO}}_{2}{-(\mathrm{OH}}^{-}{)+e}^{-}$$

(5) $${\mathrm{CoO}}_{2}{-(\mathrm{OH}}^{-}{)+C}_{6}H_{12}O_{6}\to {\mathrm{CoOOH}+\mathrm{OH}}^{-}{+C}_{6}H_{10}O_{6}$$

As it is shown GER is promoted via a redox cycle between CoOOH (or Co(III)) and CoO_2_ (or Co(IV)), producing gluconolactone (C_6_H_10_O_6_) as the final product. The redox couple CoOOH and CoO_2_ usually appears at ca. 0.6 V and 0.5 V vs. Ag/AgCl, respectively. The conversion of CoO_2_ to CoOOH ends at higher oxidation number capable of oxidizing organic molecules [20]. 

When rGO support [19] is used, its special structure that owns a lot of defects [11] enhances the electrocatalyst activity. This happens because its porous structure and the great number of functional groups, allow higher mass transfer of the OH^−^ groups derived from the alkaline electrolyte. It is characteristic that the presence of OH^−^ is necessary for the CoOOH/CoO_2_ redox couple formation and the subsequent GER progress. Moreover, it has been reported that when Co_3_O_4_ nanoparticles are included into graphene’s network, its active surface area is dramatically increased, and the adsorption of much more reactant molecules is enabled [21]. 

Doping the support with N and/or another element (P, S, etc.) is proposed as another effective strategy for increasing electrocatalytic activity of the Co_3_O_4_-based electrocatalysts and promoting GER. For example, Zheng et al. [17] reported that the coordination of the nitrogen element, with the Co metal and the reduced graphene oxide (e.g., in-situ rGO@Co_3_O_4_-NC nanomaterial), effectively promotes GER, providing a wide contact area and abundant active sites. Selectively, the glucose OH^−^ groups are attracted by the nitrogen doped-support [13], that captures easier the analyte target, while the cobalt oxides offer enough active sites for the GER process. In literature, at stronger alkaline environments, such as in 0.5 M NaOH, two pair redox couples have also been reported, at ca 0.2 and 0.5 V for the CoOOH/Co(OH)_2_ oxidation peaks and at ca 0.14 and 0.45 for the CoO_2_/CoOOH reduction peaks [22]. 

Examining GER mechanism, it can be deduced that the presence of oxidative species is essential for its promotion. However, the production of those oxidative species at neutral media, meaning values close to the blood serum pH (pH ≈ 7.2), still constitutes a challenge. When the pH of the environment lowers approaching neutral values, the OH^−^ species concentration is reduced and so many Co (or oxides) active sites remain uncovered. Thus, when GER takes place, the Co-active sites are initially oxidized at higher states, and then they are engaged into GER, reducing sensor’s response, stability, and others of its operational parameters. So, under neutral or slightly alkaline environment electrocatalyst should own increased OH^−^ adsorption ability [22]. On account of this fact the majority of the research groups focused on the exploitation of Co(OH)_x_ or Co-M(OH)_x_-based electrocatalysts. 

The presence of the redox CoOOH/Co(II) pair over Co(OH)_2_-based materials, such as Co(OH)_2_@3D graphene frameworks [23], is also important:

(6) $${\mathrm{CoOOH}+C}_{6}H_{12}O_{6}\to {\mathrm{Co}(\mathrm{OH})}_{2}+C_{6}H_{10}O_{6}$$

The high potential values cause the oxidation of the Co(II) to CoOOH which is favorable for the GER in order to be preceded. Furthermore, the combination of Co with another metal oxide, e.g., CoMn_2_O_4_/rGO (0.1 M NaOH), may offer more hydroxide species, leading to a multi-step and more complex GER mechanism [24]:

(7) $$\begin{array}{l} {\mathrm{CoMn}}_{2}O_{4}{+\mathrm{OH}}^{-}{+H}_{2}O\to {\mathrm{CoOOH}+2\mathrm{MnOOH}+e}^{-}\\ {\mathrm{CoOOH}+C}_{6}H_{12}O_{6}\to \mathrm{Co}{\left(\mathrm{OH}\right)}_{2}{+C}_{6}H_{10}O_{6}\\ {2\mathrm{MnOOH}+2C}_{6}H_{12}O_{6}\to {2\mathrm{Mn}(\mathrm{OH})}_{2}{+2C}_{6}H_{10}O_{6} \end{array}$$

The alkaline environment causes the oxidation of both metals to the CoOOH and MnOOH species (the 1st one in Equation (7)), and then each one of those, reacts with glucose molecules forming metal oxides and gluconolactone (the 2nd and 3rd in Equation (7)). In general, the bimetallic Co-M(M = Ni or Cu) electrocatalysts exhibit higher activity towards GER [18] with the metal(III) production to be imperative, as it is stated from the following proposed GER mechanism [18]:

(8) $${\mathrm{Ni}(\mathrm{OH})}_{2}{+\mathrm{OH}}^{-}↔{\mathrm{NiOOH}+H}_{2}{O+e}^{-}$$

(9) $${\mathrm{NiOOH}+C}_{6}H_{12}O_{6}↔{\mathrm{Ni}(\mathrm{OH})}_{2}+\mathrm{oxidized}\;\mathrm{products}$$

(10) $${\mathrm{Co}(\mathrm{OH})}_{2}{+\mathrm{OH}}^{-}↔{\mathrm{CoOOH}+H}_{2}{O+e}^{-}$$

(11) $${\mathrm{CoOOH}+C}_{6}H_{12}O_{6}↔{\mathrm{Co}(\mathrm{OH})}_{2}+\mathrm{oxidized}\;\mathrm{products}$$

As it is described from Equations (8)–(11), also in the case of the bi-metallic electrocatalysts the generated Ni(III) and Co(III) are the species that react with glucose and so their quantity is very critical for the progress of GER. As being reported, by many research groups [11,25,26] the key challenge to their production is the quick charge transfer between the Ni(II), Co(II) and Ni(III), Co(III), respectively, which is significantly enhanced when rGO is the support. In this direction the following GER mechanism is suggested [27]:

(12) $${\mathrm{NiCo}}_{2}O_{4}{+\mathrm{OH}}^{-}{+H}_{2}O\to {\mathrm{NiOOH}+2\mathrm{CoOOH}+e}^{-}$$

(13) $${\mathrm{NiOOH}+C}_{6}H_{12}O_{6}\to {\mathrm{Ni}(\mathrm{OH})}_{2}{+C}_{6}H_{10}O_{6}$$

(14) $${2\mathrm{CoOOH}+2C}_{6}H_{12}O_{6}\to {2\mathrm{Co}(\mathrm{OH})}_{2}{+2C}_{6}H_{10}O_{6}$$

Thus, also at this case the NiOOH and CoOOH, are the two crucial ‘species’ that promote GER [28]. When GER occurs over Cu-Co, bi-metallic electrocatalysts, for example over CuCo_2_O_4_@functionalized porous graphene, GER is also promoted due to the CoOOH and CuOOH formation [29]. On the other hand there are occasions, such as Cu-Co/rGO/pencil graphite electrode [30], that glucose electrooxidation mechanism is forwarded via the aid of CuOOH/CoO_2_ created centers. In this case GER mechanism is more complicated and is favored by the charge transfer between the Cu(OH)_2_ and CuOOH as well as between the CoOOH and CoO_2_. 

Unambiguously, rGO and porous graphene materials lower the internal resistance of the as-fabricated electrode enabling GER via the formation of redox pair peaks, corresponding to the oxidative, couples’ formation. As the resistance is low, the charge transfer becomes easier and the responsible for GER progress metal oxide couples, M(II)/M(III) or (M(III)/M(IV)) are created faster. Graphene structure manages to offer conduction channels that remain always conductive, improving GER kinetics. Moreover, as it is analyzed in the current review paper, the appropriate modification of the graphene (and its derivatives) can significantly enhance the electrocatalytic activity. 

### 1.2. Glucose Electrooxidation over Ni-Oxide Graphene (or Its Derivatives) Electrocatalysts

In alkaline environments, Ni metal is oxidized forming Ni(II) which then ‘captures’ the OH^−^ groups, producing Ni(III) that is responsible for the GER evolution as being described below [31]:

(15) $${\mathrm{Ni}+2\mathrm{OH}}^{-}\to {\mathrm{Ni}(\mathrm{OH})}_{2}{+2e}^{-}$$

(16) $${\mathrm{Ni}(\mathrm{OH})}_{2}{+\mathrm{OH}}^{-}↔{\mathrm{NiO}(\mathrm{OH})+H}_{2}{O+e}^{-}$$

(17) $${\mathrm{NiO}(\mathrm{OH})+C}_{6}H_{12}O_{6}\to {\mathrm{Ni}(\mathrm{OH})}_{2}{+H}_{2}{O+C}_{6}H_{10}O_{6}$$

More precisely, based on the mechanism described from Equations (15)–(17) the OH^−^ concentration is responsible for the Ni(II) (Equation (15)) and the Ni(III) (Equation (16)) formation. Therefore, similar to the Co (or Co oxides)-based electrocatalysts, the key element for the GER evolution mechanism and at the same time for the quick sensor response is the very high conductivity of the support along with the large available surface area [32]. This means that the support should own high surface area, to ‘host’ as many as possible active sites, and also high conductivity.

In literature, a few research groups [31,33] report the synergistic effect between the Ni nanoparticles and rGO nanosheets and how this synergy positively affects the fast charge transport, in order the redox reaction of the Ni(II)/Ni(III) couple to be continuously evolved. Moreover, they prove that the modification of the rGO support [34] can significantly ameliorates the operational parameters of a glucose electrochemical sensor. For instance, Hao et al. [34] by depositing encapsulated carbon shells with Ni nanoparticles onto the rGO surface, managed to considerably increase the number of the active centres succeeding faster electron process.

According to the GER mechanism progress over those electrocatalysts, the OH^−^ plays an important role motivating many scientific groups to develop NiO-based electrocatalysts. Thus, the graphene supported Ni-based glucose sensors operational parameters, seems to be further improved when Ni is replaced by its oxide [32,35,36,37]. 

The most common reported GER mechanism in alkaline media over NiO-based electrocatalysts is described by the following equations [32,35,36,37]:

(18) $$\begin{array}{l} {\mathrm{NiO}+\mathrm{OH}}^{-}\to {\mathrm{Ni}(\mathrm{OH})}_{2}\\ {\mathrm{Ni}(\mathrm{OH})}_{2}+{\mathrm{OH}}^{-}\to {\mathrm{NiOOH}+H}_{2}{O+e}^{-}\\ {\mathrm{NiOOH}+C}_{6}H_{12}O_{6}\to {\mathrm{Ni}(\mathrm{OH})}_{2}{+C}_{6}H_{10}O_{6} \end{array}$$

Usually the mechanism described in (Equation (18)) is expressed with one pair redox peak at ca 0.6/0.4 V vs. Ag/AgCl (by the aid of cyclic voltammetry technique) [35]. However, the poor Ni and NiO nanoparticles performance led to their combination with carbon before being deposited onto (or included into) graphene (or its derivative) structure. Especially, when carbon is appropriately modified (e.g., doped with another element), the electrode electrocatalytic performance is boosted. Zhang et al. [35] recently notified that the 2D structure of ultrathin carbon nanosheets in combination with the nitrogen-doped graphene can facilitate the electron transfer to the NiOOH (the 2nd one in Equation (18)) and then the fast adsorption of glucose molecule (the 3rd one in Equation (18)). 

Further, the transition metal oxides conductivity is improved even more when two transition metal oxides consisted of two metal cations are combined. To be specific, Dong et al. [38] deduced that the as-fabricated NiMn_2_O_4_@rGO, provide more active sites for the adsorption of glucose molecules. According to the authors, the rGO support aids much more active sites to be exposed, while when it is removed the binary-oxide aggregates forming large spheres. 

Thus, in the case of the binary oxides the production of NiOOH and second metal-OOH species, further favors the GER mechanism proceeding according to the following equations:

(19) $$\begin{array}{l} {\mathrm{NiMn}}_{2}O_{4}{+\mathrm{OH}}^{-}{+H}_{2}O\to {\mathrm{NiOOH}+2\mathrm{MnOOH}+e}^{-}\\ {\mathrm{NiOOH}+C}_{6}H_{12}O_{6}+\to {\mathrm{Ni}(\mathrm{OH})}_{2}{+C}_{6}H_{10}O_{6}\\ {2\mathrm{MnOOH}+2C}_{6}H_{12}O_{6}\to {2\mathrm{Mn}(\mathrm{OH})}_{2}{+2C}_{6}H_{10}O_{6} \end{array}$$

Initially, the binary oxide is oxidized, leading to the Ni(III) and Mn(III) formation which then they react with glucose and then are reduced back to Ni(II) and Mn(II), respectively, producing gluconolactone. Thoroughly, the support’s conductivity and the surface area are of great importance as they should ensure a continuous charge transfer between the oxidized metals. 

### 1.3. Glucose Electrooxidation over Cu-Based Graphene (or Its Derivatives) Electrocatalysts

Glucose electrooxidation reaction over graphene (or its derivatives) supported Cu (or CuO)-based electrodes have also been examined from many research groups [39,40,41]. In alkaline environments over three dimensional graphene supported Cu (or CuO), GER is generally described by the following equations [40]:

(20) $$\begin{array}{l} {\mathrm{Cu}+2\mathrm{OH}}^{-}\to {\mathrm{CuO}+H}_{2}{O+2e}^{-}\\ {\mathrm{Cu}+2\mathrm{OH}}^{-}\to {\mathrm{Cu}(\mathrm{OH})}_{2}{+H}_{2}{O+2e}^{-}\\ {\mathrm{Cu}}_{2}{O+2OH}^{-}{+H}_{2}O\to {2\mathrm{Cu}(\mathrm{OH})}_{2}{+2e}^{-}\\ {\mathrm{Cu}(\mathrm{OH})}_{2}{+\mathrm{OH}}^{-}\to {\mathrm{CuOOH}+H}_{2}{O+e}^{-}\\ {\mathrm{CuO}+\mathrm{OH}}^{-}\to {\mathrm{CuOOH}+e}^{-}\\ {\mathrm{CuO}+2\mathrm{OH}}^{-}{+H}_{2}O\to {\mathrm{Cu}(\mathrm{OH})}_{4}^{-}+2e^{-}\\ {\mathrm{Cu}(\mathrm{III})+C}_{6}H_{12}O_{6}\to {\mathrm{Cu}(\mathrm{II})+C}_{6}H_{10}O_{6}\\ C_{6}H_{10}O_{6}\overset{hydrolization}{\to }{\mathrm{gluconic}\;\mathrm{acid}\;(C}_{6}H_{12}O_{7}) \end{array}$$

According to the multi-step GER mechanism (Equation (20)), Cu is initially oxidized to CuO and Cu(OH)_2_ (Cu(II) species), which in their turn are oxidized to Cu(III) (CuOOH and Cu(OH)^4−^). The latter catalyses the adsorbed glucose molecules to gluconolactone and then to gluconic acid, while Cu(III) is reduced back to Cu(II).The GER mechanism in alkaline media (e.g., 0.1 M NaOH), over CuO/Cu_2_O-based electrocatalysts proceeds in a similar way [42]:

(21) $$\begin{array}{l} {\mathrm{CuO}+\mathrm{OH}}^{-}\to {\mathrm{CuOOH}+e}^{-}\\ {\mathrm{Cu}}_{2}{O+2\mathrm{OH}}^{-}{+H}_{2}O\to {2\mathrm{Cu}(\mathrm{OH})}_{2}{+2e}^{-}\\ {\mathrm{Cu}(\mathrm{OH})}_{2}{+\mathrm{OH}}^{-}\to {\mathrm{CuOOH}+H}_{2}{O+e}^{-}\\ {\mathrm{CuOOH}+C}_{6}H_{12}O_{6}\to {\mathrm{Cu}(\mathrm{OH})}_{2}{+C}_{6}H_{10}O_{6}\\ C_{6}H_{10}O_{6}\to C_{6}H_{12}O_{7} \end{array}$$

In more neutral environments, such as in PBS (pH = 7.4), GER is evolved via the formation of Cu (I)-glucose complex [43] which then releases one proton for forming gluconic acid (usually at 0 V vs. Ag/AgCl) while at the same time the reduction of the formed Cu(II) (at−0.25 V vs. Ag/AgCl) takes place.

In case of a binary Cu-based electrocatalysts, namely CuCo_2_O_4_, glucose electrooxidation proceeds as follows:

(22) $$\begin{array}{l} C_{6}H_{10}O_{6}\to C_{6}H_{12}O_{7}\\ {\mathrm{CuOOH}+C}_{6}H_{12}O_{6}\to {\mathrm{Cu}(\mathrm{OH})}_{2}{+C}_{6}H_{10}O_{6}\\ {\mathrm{CoOOH}+C}_{6}H_{12}O_{6}\to {\mathrm{Co}(\mathrm{OH})}_{2}{+C}_{6}H_{10}O_{6} \end{array}$$

Analysing GER mechanism leads to the realization that the sensing properties of enzymeless glucose electrochemical sensors, such as sensitivity and detection limit, totally depend on the route of the mass and charge transport between M(II)/M(III) or M(III)/M(IV). Thus, the key-requirement is this route to be of low internal resistance and at the same time to offer the capacity for as many as possible active sites. Graphene and especially reduced graphene oxide are considered [42,44,45,46,47] of the most appropriate materials to use as support since they facilitate the required electron transfer among the reaction sites so that the redox pair M(II)/M(III) or M(III)/M(IV) to be created continuously for promoting GER. Their superior electric conductivity, large surface area, as well as their biocompatibility facilitate GER. However, the offered electron pathways from graphene (or its derivatives) and the active surface areas totally depend on its synthesis route method [48]. 

## 2. Graphene-Based Nanomaterials for Glucose Electrochemical Sensors

### 2.1. Direct Growth (or Deposition) of Graphene (or Its Oxides) onto Metallic Substrates

Graphene is usually used independently serving as support to metallic nanoparticles. However, recently in the pursuit of more stable electrochemical glucose sensors, direct growth of graphene nanosheets onto conducting substrates is suggested. The higher advantage of this synthesis method is the omission of the placement step of the graphene-based nanoelectrode onto the electrode’s substrate. In such a way there is not any risk of destroying graphene’s structure, thus the cost lowers and this is a timesaving procedure [49]. Furthermore, the direct growth of graphene leads to an induced-defect 3D structure that minimizes the internal resistances, therefore increases sensor’s response. The created defects are desirable for achieving higher mass transport of the analyte and of the charge, which is required according to our analysis of GER mechanism.

Currently, according to our literature research, the CVD synthesis is suggested as the most appropriate method for the graphene direct growth onto metallic substrates [50,51,52]; due to its reproducibility and the high-quality graphene-based electrodes that gives. Following a CVD synthesis route single or multilayer graphene with higher surface area can be produced. Moreover, by varying the experimental conditions the produced graphene layer can be grown onto various substrates giving a layer with a great uniform thickness. 

**Figure 1:**
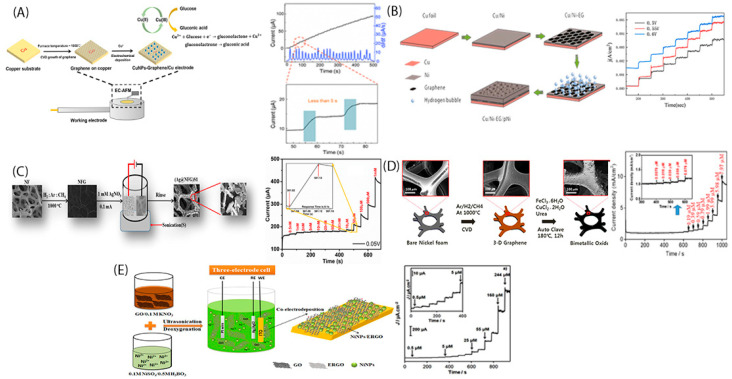
(**A**) CuNPs-graphene/Cu bioelectrode fabrication steps and its amperometric response to glucose injection [50]; (**B**) Synthesis procedure of Cu/Ni-EG/pNi, and the respective amperometric response to glucose injection [53]. (**C**) TEM images and amperometric response of Ag vertically deposited onto 3D nickel foam graphene and its amperometric response to glucose injection [54]; (**D**) Synthesis procedure of the CuFe-O/graphene onto nickel foam substrate and its amperometric response to glucose injection [41] and (**E**) Ni^2+^ and Co co-reduction onto ITO for the Ni/rGO/ITO synthesis and its amperometric response to glucose injection [32].

Recently, Wang et al. [50] following the CVD method, they initially grown a graphene layer onto a Cu substrate and then via the electrochemical deposition technique they placed Cu nanoparticles (Figure 1A). The as-resulted electrode was directly used for glucose sensing. They reported a wide linear range of 0.02–2.3 mM, a low detection limit (1.39 μM, S/N = 3), good sensitivity (379.31 μAmM^−^^1^cm^−^^2^), excellent selectivity and quick response (<5 s). The good sensitivity and stability were attributed to the full coverage of the bilayer graphene from the electrodeposited monolayer of the Cu nanoparticles (Figure 1A). The uniformly dispersion of Cu nanoparticles onto graphene film highly increased the electrodes conductivity. Additionally, the growth of thin graphene layer onto the metallic substrate increased the electron transfer rate between the platform and the catalyst, which is necessary for the quick response of a sensor. 

The direct growth onto metallic substrate graphene-based electrodes should achieve, optimum mass, and charge transport for accurate and quick glucose sensing. This is a challenge that should be overcome. Therefore, the internal electrode resistances should be reduced as much as possible. Towards this direction, a few research groups suggested the Ni foam intervention, which could play either the role of the electrode skeleton [53] to deposit other materials or the role of the substrate [54]. This suggestion was based to the fact that theoretically, the contact resistance between graphene and Cu foil is higher than graphene and Ni [55]. Graphene nanosheets and nickel foam are two materials of different thermal expansion whose combination can lead to the formation of many deficiencies (wrinkles and ripples), which can extremely expand the surface area, onto where metal nanoparticles can be deposited.

In an attempt to increase such electrodes conductivity, Zhaodi et al. [53] suggested the use of a Ni layer as a middle layer before the graphene growth process takes place. Alternatively, they used graphene as bottom layer for the Ni deposition via a hydrogen bubble template method (Figure 1B). The middle Ni layer reduced the Ni pores size resulting in higher electrochemical surface area; as well as the internal resistance resulting in higher current response. According to their results, they succeeded a high sensitive glucose electrode (6161 μAmM^−1^cm^−2^) (Figure 1B). 

Since Ni foam has large pore size can be used also as substrate [41,54,56] for direct growth of graphene onto it. This synthesis strategy significantly reduces electrode’s resistance, increases electron transfer ability, and lacks binding elements which reduce the electrodes activity. Namely, the use of Ni foam as substrate for the graphene growth via a CVD synthesis route was followed by Usman et al. [54]. The research group following a CVD synthesis method and using nickel foam as substrate and CH_4_ as carbon source, managed to fabricate a glucose electrode with a very high porosity (Figure 1C) ensuring a very high surface area for easy analytes mass and charge transport. Then, they vertically deposited Ag nanoparticles onto graphene/Ni foam, exploiting the whole offered 3D surface area. The vertical deposition also led to a further reduction of the electrode’s internal resistance and to a significant increment of the electrode conductivity. Specifically, they achieved an ultra-high sensitive glucose electrode (2 × 10^11^ μAmM^−1^cm^−2^), (Figure 1C). 

Accordingly, Figure 1D shows the direct growth of graphene onto Ni foam substrate, via a CVD synthesis method and the subsequent growth of the metal oxide (CuFeO) via a hydrothermal reaction, suggested by Jeong et al. [41]. The special Ni foam structure encouraged the formation of defects on the three-dimensional graphene which are advantageous for the metal crystal seeds self-assembly. The morphology and the surface area of the as-produced electrode was controlled by the salt precursor parameters. The CuFe-O/graphene/nickel foam electrode exhibited very high sensitivity (368 × 10^3^ μAmM^−1^cm^−2^) and 0.0079 μΜ limit of detection. The electrode’s so high sensitivity was attributed to the porous and interconnected networks in combination with the lack of binders during its synthesis procedure, which dramatically decreased the internal resistance. 

However, the direct growth of graphene onto metallic substrate electrode encloses a possibility the graphene to be contaminated with metallic impurities [57], influencing its electrochemical and electronic properties. So, different metallic conductive substrates, such as indium tin oxide (ITO), fluorine tin oxide (FTO) [58] and others have also been studied [32,59]. 

Chen et al. [59] reported the direct growth of graphene onto Cu foil via CVD method, its spraying with poly(methyl methacrylate, PMMA) and then the PMMA/graphene transfer (via a wet transference process) onto the ITO. However, we deduce that this synthesis method reintroduces the transfer step that was disregarded according to the direct growth of graphene onto metallic foil. Despite this disadvantage the growth of graphene onto ITO, stabilized the electrode performance, enhanced its conductivity, and remarkably increased the electrodes sensitivity from 289.8 to 466.1 μAmM^−1^cm^−2^. 

Alternatively and for avoiding the transfer step, Urhan et al. [32] proposed the direct deposition of rGO and metallic nanoparticles onto an ITO surface, via electrochemical co-reduction of GO and Ni^2+^ onto ITO, applying a constant potential and for a specific time duration (Figure 1E). The as-fabricated electrode was notably of very high sensitivity, 185.2 × 10^3^ μAmM^−1^cm^−2^ exhibiting also very low limit of detection (40 nM glucose) (Figure 1E). The uniform dispersion of Ni nanoparticles along with the lack of a lot of chemical reagents, lowered the electrode internal resistance offering a larger contact area between Ni nanoparticles and the rGO. That way, the charge transfer between the redox pair M(II)/M(III) and glucose molecule is accelerated and therefore sensor’s sensitivity is enhanced. 

Thus, in the case of the direct growth of graphene-based electrode onto a substrate, the synthesis method that is used, strongly depends on the kind of the substrate. The use of a Cu foil for example, allows the direct growth of the graphene mostly via a CVD method. The CVD method when includes Ni foam as an incidental element seems to reduce the electrode’s internal resistance, producing electrodes of much higher sensitivity. Based on this, graphene can be directly grown via a CVD method on a Ni foam substrate, which plays the role of a 3D skeleton of the electrode, giving also remarkable results as far as concerns the electrode’s sensitivity. However, the use of a different than metallic substrate requires the initial growth of graphene directly onto the metal substrate and then its subsequent transfer to the desired non-metal substrate. That means that there is great risk of damaging graphene’s structure. Thus, in order this transfer step to be avoided, but also an alternative substrate to be used, a different synthesis method should be adopted; for example, the direct co-reduction of GO and metallic ions onto a substrate. 

### 2.2. In-Situ Growth of Metallic Nanoparticles into Graphene Nanosheets

The use of graphene material as support enhanced the electrocatalytic activity, of the non-precious metals, receiving a lot of attention for glucose sensing [8,12,60]. However, the uniform dispersion of them onto graphene support, without increasing the electrode’s internal resistance and at the same time acquiring a high catalytic performance, remains a challenge. According to many research groups [22,31,42,61,62,63,64,65,66], an in-situ growth of the metallic nanoparticles inside graphene (or its derivatives) matrix, ensures homogeneous distribution of the nanoparticles and enrichment of the electron transport efficiency. Therefore, the formation of an inside nanostructured matrix with graphene (metal-graphene matrix composites), enhances significantly the electrocatalytic activity towards GER. 

One of the most common approaches that is adopted when in-situ fabrication synthesis route is followed, is the application of metallic organic frameworks (MOFs) to the role of the precursor. The MOFs are being formed by the combination of inorganic element (metal element) being surrounded by organic linkers. Specifically, they own a cage-like structure which imparts its crystalline porous structure and an extremely huge internal surface area. Those special characteristics along with their functionable structures, chemical tunability and high compatibility, have make MOFs very attractive materials for a variety of applications [67]. 

The MOFs involvement [17,29,61,62,65,68] and the exploitation of their metal active sites, into the fabrication of electrochemical sensors development, suggest improved characteristics attributed to combination of their porous structure with a conductive material, such as graphene. In the context of exploring the potentials of MOF-derived graphene-based nanomaterials as electrodes for electrochemical sensors, some groups employed stabilizers (e.g., poly(vinylpyrrolidone), in order to achieve uniform stabilization of the metal nanoparticles into graphene network. The intrusion of such stabilizers forms a very good interface, boosting the as-developed sensor’s sensitivity [42]. When 2D MOF ultrathin sheets are in-situ fabricated with graphene and metal nanoparticles; mass and electron transfer are higher, and so glucose electrooxidation reaction is further promoted. Additionally, the exfoliated graphene contributes positively to a MOF synergistic effect, structure, and the total performance.

**Figure 2:**
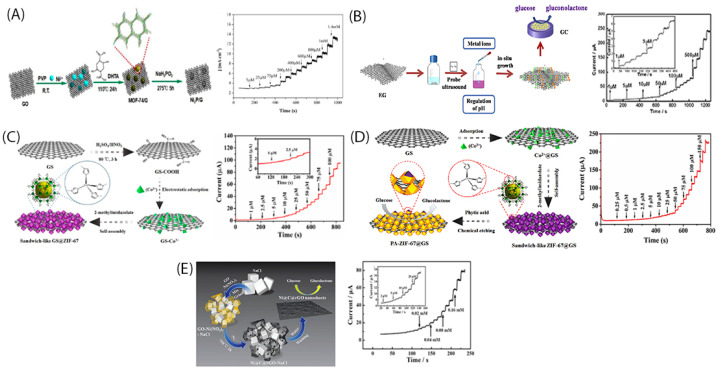
(**A**) In-Situ synthesis procedure of Ni_2_P/graphene and the respective amperometric response to glucose injection [61]; (**B**) In-Situ synthesis process of a NiCo-MOF-exfoliated graphene electrocatalyst and the respective amperometric response to glucose injection [62]; (**C**) In-Situ synthesis of graphene@ZIF-67 heterostructure and the respective amperometric response after the glucose injection [63]; (**D**) In-Situ growth of MOF into physically exfoliated graphene, functionalized the used MOF (ZIF-67) using phytic acid [70] and (**E**) In-Situ synthesis Ni@C@rGO nanosheets and the resulting amperometric response to glucose detection [34].

Taking as an example, Figure 2A depicts an in-situ Ni_2_P growth into graphene process, suggested by Zhang et al. [61]. Initially, GO was obtained with Hummer’s method, and then it was mixed with a water-soluble polymer (PVP) for avoiding GO aggregations. The utility of polymers is extensively discussed to the next sub-section. After, they added the metal salt and the MOF-74/G, aiming at the coordination of the metal ions with the GO via the PVP. This coordination facilities the uniform nucleation of the MOF onto GO films. Finally, the GO sheets transformed to graphene ones via a hydrothermal process. The sensitivity of the as-suggested electrode was estimated 7234 μAmM^−1^cm^−2^ and the limit of detection, 0.44 μM. 

As the authors report in this case graphene material played a dual role; as they enhanced both NiP nanoparticles activity and stability, while at the same time increased MOFs electrical conductivity and mechanical stability. However, this is a time-consuming method, consisting of multiple steps and depends on many chemical reagents, whose amount, and the technique that they will be utilized, play a great role to the resulting electrocatalyst’s properties. Thus, at this point arises the issue of the reproducibility of the above synthesis method. 

According to some research groups the defects and edge planes of exfoliated graphene (EG), can enhance electrochemical glucose sensor sensitivity. It is characteristic that in-situ MOF-exfoliated graphene could promote quick heterogeneous electron transfer of redox molecules, conferring a very low M(II) redox potential, thus accelerating GER. Moreover, it should be mentioned that sometimes exfoliated graphene is chosen instead of graphene, because is more soluble to some reagents.

A characteristic example of in-situ growth of 2D MOF onto electrochemical EG surface synthesis method is depicted in Figure 2B [62]. Aiming at developing a high sensitive and stable NiCo-MOF-EG electrode for glucose sensing, Liu et al. [62] followed an in-situ strategy. The MOF nucleation happened preferentially giving very thin nanosheets, as it is shown in Figure 2B, via an easy and fast procedure. The special 2D MOF interconnected porous structure offered a very high specific surface area which facilitated the electrons transfer via metal ion to glucose molecule. 

Since graphene material plays the crucial role to the final glucose electrodes operational characteristics, some research groups further explored the effect of the production method of exfoliated graphene to an in-situ synthesis method. Particularly, Chen et al. [63,69] and other researcher groups [62,70] in a series of publications stated the advantages of an in-situ synthesis method of physically exfoliated graphene in combination with MOFs. Remarkably, physically exfoliated graphene presents lower background current and excellent electron transfer ability; two special characteristics that an electrochemical sensor should own for having high performance. 

Figure 2C describes the synthesis strategy of in-situ growth of MOF (ZIF-67) into physically exfoliated graphene nanosheets. The physically EG was subjected to a pretreatment procedure and then was mixed with PVP and the respective metal salt. Then other reactant molecules, methanol and methylimidazole were added. The same procedure was followed for the pure ZIF-67 preparation without adding the graphene. The usage of both surfaces provided more geometric area for the in-situ grown metallic nanoparticles and consequently more electrocatalytic active sites. In this case polyhedral Co-based zeolite imidazole frame was used as precursor (MOF); and the as-suggested method led also to hierarchical structure which further improved the catalytic activity of the metallic nanoparticles [63]. According to their results, the sensitivity was estimated at 1521.1 μAmM^−1^cm^−2^ and the limit of detection equals to 0.36 μΜ. 

Making one step further, Sun et al. [70] fabricated a sandwich-like functionalized hybrid electrocatalyst. To be more specific, after the in-situ growth of MOF into physically exfoliated graphene, they functionalized the used MOF (ZIF-67) using phytic acid. The MOF functionalization took place via a controlled chemical etching process with the phytic acid decorated ZIF-67 particles to have a core-shell structure (Figure 2D). Thus, an ultrasensitive (2718.3 μAmM^−1^cm^−2^) non-enzymatic electrochemical glucose sensor was achieved. In general, the functionalization process of the MOFs with an etching reagent can lead to further expose of the metal active sites, making available more electrochemical active surface area. 

The in-situ fabrication synthesis strategy using MOF as sacrificial element certainly improves electrodes sensing performance. However, the controllable synthesis (e.g., for acquiring hierarchical structure) as well as the development of a highly efficient interface for fast charge transfer, constitutes a great challenge. Currently, the MOF functionalization seems to enhance this interface, meanwhile much further research is necessary. 

The in-situ synthesis method has also been examined for carbon incorporation into a graphene sheet to ameliorate electrocatalyst glucose sensing performance. The inclusion of carbon material increases more the pore volume and the specific surface area. Hao et al. [34] for example followed an in-situ encapsulating and anchoring strategy using GO as carbon source for the development of Ni@C@rGO electrocatalyst. In detail, they fabricated ultrathin carbon shells around the metallic nanoparticles (Ni) anchored on the rGO nanosheets. The rGO nanosheets were formed by GO pyrolysis, while under the same pyrolysis process the Ni precursor formed the Ni metallic nanoparticles which homogeneously anchored onto the rGO surface (Figure 2E). The GO presence affects the catalytic growth effect of Ni nanoparticles and therefore is responsible for the ultrathin carbon shells formation around the Ni nanoparticles. The inclusion of salts, such as NaCl played a vital role to prohibit graphene aggregation. The respective sensitivity was calculated 1211.41 μAmM^−1^cm^−2^ and the limit of detection 0.34 μM (Figure 2E). 

The as-proposed synthesis method, leads to much larger specific area, as the metallic nanoparticles can be highly dispersed; while the ultrathin carbon shells in combination with the rGO high conductivity, are responsible for the very quick electron transfer. Moreover, a better dispersion leads more metallic nanoparticles to be anchored onto the support and so more active sites are available to glucose molecule and the electrochemical sensor’s sensitivity is increased [71]. 

In general, the in-situ fabrication techniques seem to help avoid agglomeration phenomena, improve metal nanoparticles catalytic activities, and increase the specific surface area. Nevertheless, the degree of synergy between the in-situ fabricated element (MOF or carbon) and the graphene (or its oxides) nanosheets seems to be the key-requirement for a high electrocatalyst glucose sensing performance. Consequently, since the type of the in-situ element along with other chemical reagents that are involved into the synthesis procedure define the synergy, this synthesis method should thoroughly be explored. 

### 2.3. Laser-Induced Graphene-Based Electrode Synthesis and Functionalization

Laser induced method for graphene synthesis and functionalization is very promising [72]. Using laser tools, the synthesis and functionalization processes are accurately controllable, cost much less than other methods that use chemical agents, also allow the direct growth and functionalization of graphene onto the desired substrate. Moreover, laser methods have great reproducibility since the synthesis process is controlled by the laser operational characteristics, such as wavelength, energy density, etc, and not by chemicals. One way that has been followed by research groups [73] to overcome this challenge is the creation of surface (or structural) defects using more controllable techniques, such as with the use of laser. 

It has been found [74] that under the appropriate functional conditions, the laser-engraved defects can be in balance with electrical conductivity. Thus, the created channels and pathways make much easier the charge and ions transport leading to faster response and higher sensitivity of a sensor [75]. Another important advantage of the laser induced graphene electrodes, is the on-site construction of them onto flexible substrates/platforms; in combination with the fact that the raw materials are of lower cost without requiring any post-treatment steps, it seems to gain significant ground as a synthesis strategy. Following this synthesis method the degree of functionalization and the functional groups (-OH, -COOH and others) that are created depend on the operational characteristics of laser, such as wavelength, energy density, etc., [72]. 

Laser induced graphene from polymers (or known LIG) seems to thrive as a process since its discovery in 2014 [72]; while it seems to be explored for the development of glucose electrode sensors in the last few years [76]. More specifically, graphene can be selectively formed through a carbonization process induced by the laser irradiation [77], while the N and O-groups of the polymeric substrate are decomposed and when the produced gas is also used, a 3D porous structure is obtained. The remaining nitrogen and oxygen can provide sites for functionalization, that as being mentioned in the current review paper, are very critical for the evolution of glucose electrooxidation reaction [78]. Moreover, the resulted LIG dependent on the operational laser conditions, owns numerous graphene edges which are desirable for its further functionalization. 

Commercial polyimide (PI) polymer is one of the most common graphene sources that is explored for LIG electrode development. The graphene’s hexagonal structure is created via subsequent carbonization and graphitization processes, due to the aromatic sp^2^ carbons of PI [79]. However, the technique of metallic nanoparticles addition to LIG structure is still under investigation. One of the main challenges that should be overcome is the anisotropic conductivity of the LIG 3D structure, which dependent on the preparation method that is followed, could affect the preparation time and the uniformity of the metallic nanoparticles or layer onto graphene surface. Namely, it has been found that electroplating deposition of metallic nanoparticles is acceptable only for large LIG electrodes [80], while otherwise it may the deposition time be increased dramatically leading to a non-uniform deposition. So, currently most of the efforts are focused on the exploration of uniform metallic nanoparticles deposition or inclusion of them into the LIG structure, aiming also at its conductivity increment. 

At present, electroless processes are reported as appropriate alternative methods for ensuring homogenous deposition of metallic nanoparticles onto LIG or even the creation of metallic layer onto it. Substrate-assisted electroless deposition method, developed by Qu et al. [81], is a facile method for depositing metallic nanoparticles onto a carbon-based support material, in absence of any reducing agent. This is succeeded by placing the support onto a metallic substrate which has redox potential lower than that of the metal ions that will be reduced to metallic nanoparticles, without the metal ions redox potential to be required to be higher than carbon’s (as common electroless deposition requires). 

**Figure 3:**
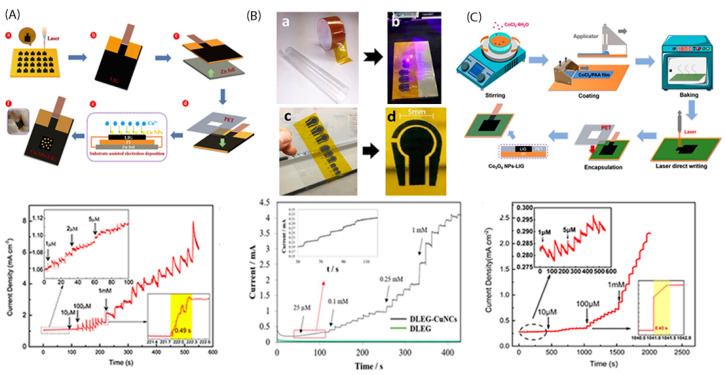
(**A**) Cu NPs-LIG synthesis procedure and the respective amperometric response to glucose injection [39]; (**B**) Direct laser engraved graphene (DLEG) decorated with pulse deposited copper nanocubes and the respective amperometric response to glucose injection [82] (Taping Kapton tape on a thin sheet of PVC (a). Direct laser reduction of the Kapton tape to graphene forming sensor electrodes (b,c). A DREG 3-electrode platform, prepared for further modification (d).) and (**C**) LIG assisted encapsulation of Co_3_O_4_ nanoparticles synthesis method and the respective amperometric response to glucose injection [83].

Characteristically, recently, Zhang et al. [39] constructed a 3D porous LIG (with flake shape) electrode deposited onto a flexible substrate for glucose detection, having PI as polymeric substrate and using the produced gas through carbonization process (Figure 3A). Then, Cu nanoparticles were deposited onto LIG by a substrate-assisted electroless deposition process [81]. The resulting graphene nanosheets owned crumples and curves along with small holes, while a lot of graphene edges were present at the laser induced substrate. The graphene ‘defects’ were the channels for the electrons (being provided by the metal) delivery via the metal-carbon ‘bond’ and so the key for glucose electrooxidation reaction evolution. The sensitivity of the Cu-LIG nanoelectrode was estimated 495 μAmM^−1^cm^−2^ and the detection limit, 0.39 μM (Figure 3A). 

Consequently, the LIG, in flakes shape, was successfully functionalized with metallic nanoparticles, creating a small sensor electrode area, avoiding the use of so many chemical reagents, comparable to the other synthesis methods, described in the current review. Cu deposition nanoparticles (cubic shape) process lasted only several minutes, ensuring at the same time a very great homogenous deposition of them onto the LIG surface.

Thus, two factors (linked to the synthesis method) that affect the sensitivity and other operational parameters, of a non-enzymatic LIG-based electrochemical glucose sensor, are tracked. The first one is the method of the metallic nanoparticles or metallic coating layer addition to the LIG that will ensure homogeneous placement and so raise of the LIG conductivity, while the form of the LIG (foam, ribbon or flakes), which essentially should be further explored, is the second one. 

Zhu et al. [84] followed an electroless plating process, for coating Ni and Ni/Au layers onto LIG fibres and foams, that were produced via a PI substrate. As it was expected the plating time is a very crucial parameter for the coating of the LIG with the appropriate metallic layer thickness that will not block its pores. Furthermore, the porous structure presence facilitates the reactants diffusion, leading to higher sensitivity and faster sensor’s response. Another important parameter was that the Ni plating took place preferably onto LIG and so there was no need for using special cover to outline the working region of the process. Additionally, they confirmed that the LIG shape could affect sensors sensitivity, since the LIG fibers presented almost four times higher sensitivity (3500 μAmM^−1^cm^−2^) (compared to foams) to glucose detection. 

According to literature the sensitivity of the LIG electrodes seems to be further enhanced when is double sided used. Specifically, the available to reactants surface area is much larger, leading to very high sensitivity values and very low detection limit. The research work of Tehrani et al. [82] is a representative example, reporting 4532 μAmM^−1^cm^−2^ glucose sensitivity and 250 nM low detection limit (Figure 3B). The LIG source was a Kapton tape (Figure 3B) that was engraved to its both sides and then Cu nanocubes were deposited onto both sides by pulse electrodeposition technique. Moreover, the as-fabricated [82] glucose sensing platform exhibited a great stability, as after one month of repeated experiments the stability rate was estimated higher than 98%. 

Thus, since the LIG electrodes give very promising results to electrochemical glucose detection, the researchers have started searching methods for ameliorating its characteristics. In-situ growth of metal nanoparticles into support matrix constitutes a strategy that as we highlighted in our previous sections, ameliorates electrochemical glucose sensors operational characteristics. Thus, the LIG can also be further modified by embedded metal nanoparticles into its matrix. Zhao et al. [83] reported an in-situ Co_3_O_4_ nanoparticles into LIG matrix synthesis method according to which, before laser application, the metallic nanoparticles are homogeneously dispersed into the liquid polymeric precursor, then the formed ‘pasta’ covers the coating and after the laser is applied, as shown in Figure 3C. This strategy aids the metallic nanoparticles being incorporated homogenously into the LIG matrix, avoiding any agglomeration. The sensitivity of the as-fabricated electrode was reported 214 μAmM^–1^cm^–2^ and a very low limit of detection (LOD) of 0.41 μM, indicating that the process needs further improvement.

Under this background, the design of versatile and facile strategy for fabrication of flexible, shape-controllable metal-based LIG electrodes on various substrates and shapes, needs further exploitation. Laser direct techniques are simple, convenient, and of low cost, but are currently applicable to customize PI substrate mainly, while the metallic elements are electrodeposited onto the LIG after the laser engravement. However, metallic heterogeneous polymer precursors can be used to in-situ incorporate metal oxide nanoparticles, but this method is at premature stages. 

### 2.4. Polymer Functionalized Graphene-Based Electrodes

Graphene functionalization is a strategy that aims at its surface modification for altering graphene’s chemical and physical properties, mainly reducing the cohesive forces that are deployed between the sheets [74]. This is separated into two main categories, non-covalent and covalent functionalization [75]; meaning that in the first case covalent bonds are created, while in the second one the bonds are related to Van der Waals forces. 

Graphene as material displays aggregations through Van der Waal interactions, and when defects and functionalities (such as oxygen groups) are in excess, its conductivity and consequently electrocatalytic activity of the graphene-based catalyst, are negatively affected. Therefore, for eliminating such phenomena, organic conducting polymers (with very good electrical conductivity) are used as graphene modifiers and via non-covalent bonding they stabilize graphene’s structure. 

In literature [76] covalent functionalization of graphene has extensively been investigated and has been related to electrons sunder in applications, such as in the electrochemical sensors, where the quick transport of electrons is important. However, such phenomena are not observed with polymer-based graphene nanoelectrodes. Furthermore, in comparison to covalent, the non-covalent functionalization is considered much more efficient as graphene keeps its electronic properties.

One of the first strategies that was proposed, in order the properties of the functionalized graphene, to be improved; included the dilution of the desired polymer into the graphene oxide solution, before taking place graphite oxide’s reduction reaction process. In that way the resulted polymer-graphene material acquires higher solubility, very good conductivity and offers much larger area that can accommodate much more metal nanoparticles, lacking any aggregation trends. Moreover, the as-resulted pasta can be applied directly to a glassy carbon electrode for further experimental investigation [85,86]. 

In the case of glucose electrochemical sensors, the combination of polymer-graphene and metal oxide [87] increases significantly its sensitivity along with its stability. Those improved characteristics of the glucose sensors are attributed to the introduced polymer element that increases more the graphene’s conductivity. The higher graphene conductivity allows the faster electron mobility among the catalyst and reactants. Moreover, the non-covalent bonding between the graphene and a conducting polymer prevents the last one from aggregating. The lack of aggregation leaves more disposable area for the deposition of much more metallic nanoparticles and therefore much more catalytic active sites. The synergistic effects between the polymer/graphene and metallic nanoparticles enhance even more the sensor’s performance. 

**Figure 4:**
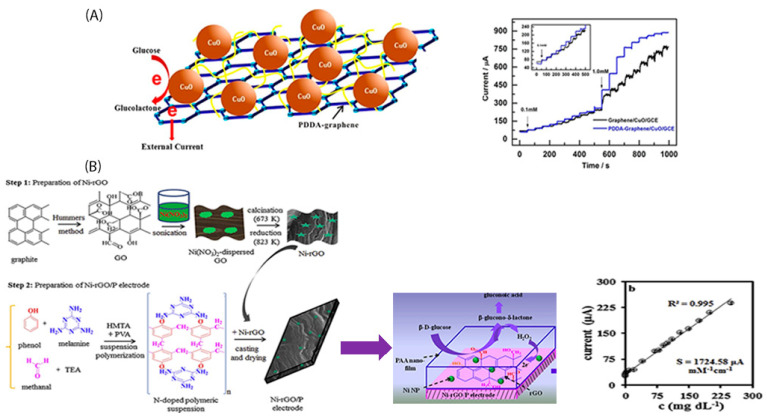
(**A**) Poly-(dimethyl diallyl ammonium chloride (PDDA) functionalized graphene decorated with CuO nanoparticles and its respective amperometric response to glucose injection [87]; and (**B**) PAA covered Ni-rGO synthesis procedure (left) and its respective amperometric response to glucose injection [90].

Figure 4A shows the structure of a poly-(dimethyl diallyl ammonium chloride (PDDA) functionalized graphene with CuO nanoparticles and the respective amperometric response, to glucose detection. According to catalyst synthesis process the PDDA polymer, was firstly homogenously dispersed into the graphite oxide solution and then followed the addition of the reducing agent which led to the reduction of graphite oxide to graphene. Its high sensitivity (4982.2 μAmM^−1^cm^−2^) and the low detection limit (0.20 μM) were attributed to synergistic effects as well as to the large area that PDDA-graphene offered to metal oxides [87]. Another noteworthy asset of this kind of electrode was its successful testing under human serum without the need of any pretreatment step.

The amount of the polymer that is added is a very crucial key as determines the characteristics of the final polymer/graphene catalyst. An excessive amount of polymer, could obstruct the catalytic sites, leading to current response decrease, while a lower amount could lead to a lower current response than the maximum that the catalyst could give. 

According to the most recent works, rGO seems to have better behaviour than graphene when it is combined with polymers; as rGO, on the contrary to GO, is more soluble to polymeric solutions [88]. Moreover, as it was above-discussed in previous sections, the rGO as a 2D monolayer hinders the agglomeration and offers conductive channels, leading to ameliorated glucose sensors characteristics. Especially when rGO is produced from the exfoliated GO reduction, the inclusion of a polymer into the synthesis method ameliorates glucose sensor’s characteristics. Therefore, the rGO dispersing ability led to the exploration of various novel synthesis methods of polymer-rGO nanocatalysts for glucose detection.

Under this background, Deshmukh et al. [89] explored the functionalization of reduced graphene oxide with polyaniline (PANI) that is a hydrophilic polymer [89]. For the PANI/rGO formation the researchers followed the same synthesis strategy. They initially made a fine polymer/rGO suspension and then they proceeded to electrochemical synthesis method (cyclic voltammetry) for acquiring the PANI/rGO. The two materials (PANI and rGO) formed non-covalent bonds; and then the PANI/rGO was decorated with Ag nanoparticles. The Ag dispersion onto the PANI-rGO increased the electrode roughness enabling at the same time the adsorption of more analyte molecules and so amplifying electrochemical sensor’s current response. The current response measurements show 2766.4 μAmM^−1^cm^−2^ sensitivity and 0.79 μM low limit of detection. Despite that the as-prepared electrode did not be examined under real samples, the sensitivity was estimated at around neutral pH values, indicating its good electrocatalytic characteristics even at close real human environmental conditions. 

The choice of the appropriate polymer material seems also to be a really very important key parameter that strongly affects a glucose sensor performance. Taking as an example the work of Deshmukh et al. [89], the N-atoms of PANI seems to engage with the metal nanoparticles, enabling electronic interaction and in combination with rGO large surface area, enhance electron transfer and so glucose electrooxidation reaction process. Furthermore, according to Apátiga et al. [86] work, the polymers with aromatic ring structure, when they are dispersed into graphene solution, due to the π-π graphene stacking, the so long-term dispersion interactions prevail. On the contrary when the polymers’ structure is linear, prevail the short-range dispersion interactions, that are attributed to the CH-π stacking. 

PANI has been proved to be a very safe choice for compose polymer-graphene electrodes for enzymeless glucose sensors and so a few research groups [54,91,92] investigated its feasibilities. However, its use has been extensively investigated mostly in enzyme-based glucose fuel cells, showing encouraging results and so there is plenty room for exploration of it into enzymeless electrochemical glucose sensor’s field. 

Notably, Bairagi et al. [90] suggested a novel synthesis method of an enzymeless glucose electrode using a conductive polymeric substrate instead of a carbonaceous one, including also metal nanoparitcles/rGO-polymer into its composite. Following an electro-polymerization technique, they managed to develop a polyacrylamide (PAA) film (polymeric substrate) by AA monomers polymerization on a Ni/rGO/P electrode. 

So, in this work into the metal-rGO-polymer catalysts synthesis method, we recognize the combination of three different ways that have been adopted to increase the total electrode conductivity, mostly: The first one is the homogeneous dispersion of the metal precursor into the GO (produced by Hummer’s method) solution before GO calcination and reduction (in-situ synthesis method). The second one includes the use of melamine, among the polymeric reactants, whose N-atoms (as in case of PANI) increase electron conductivity and the third one is the addition of the Ni-rGO into the polymerization synthesis procedure of the Ni-rGO (Figure 4B). Then a PAA layer was developed by ascorbic acid electropolymerization via CV technique. The addition of the polyacrylamide as a top layer onto the Ni-rGO-polymer catalyst, dramatically increased the electrochemical active surface area, leading to a really highly sensitive [93] electrochemical glucose sensor (1724.58 μAmM^−1^cm^−2^) and low detection limit equal to 0.0126 mgdL^−1^ (Figure 4B). Furthermore, the metallic nanoparticles are observed to play the role of the catalyst to the GO reduction as well as to the PAA layer formation and the role of the electrocatalyst to glucose sensing. 

In aqueous solutions the PAA polymer produces carboxylate anions that can bind with metal cations (e.g., Pd^2+^ [94], Cu^2+^) before the reduction process and the deposition of metal nanoparticles, take place. In that way the GO aggregation is reduced and the metal nanoparticles dispersion on the GO surface is increased. Therefore, the key to further improve GO or rGO dispersion is the increment of the interfacial interactions. Towards this target a few synthesis strategies have been proposed, with the homogeneous dispersion of the polymer into the solution before the GO or rGO formation takes place, to be the most common one. For the GO or rGO production the electrochemical methods seem to be preferred. 

In summary, the development of polymer-graphene based electrodes is at very primitive stages, and so many synthesis methods in the future will be proposed and explored. Conducting polymers’ morphology and structure offer the possibility for using their functional groups in various synthesis methods. Additionally, some of them have chemical stability along with tunable conductivity and are of low cost, as well.

Unfortunately, the dispersion ability of graphene (or its derivatives) along with the appropriate polymer is a key parameter that strongly affects the final properties of the polymer/graphene based electrocatalyst. The polymer introduction into the synthesis method, may improve GO or rGO dispersibility, but could have a negative impact on sensors properties if they are not selected carefully. 

## 3. Challenges and Future Perspectives 

According to the current research, different synthesis strategies can be applied to ameliorate an enzymeless graphene-based glucose electrochemical sensor’s characteristics. Among them we recognized that the suggested methods could be classified into four main categories: (i) direct growth of graphene (or oxides) on metallic substrates, (ii) in-situ growth of metallic nanoparticles into graphene (or oxides) matrix, (iii) laser-induced graphene electrodes and (iv) polymer functionalized graphene (or oxides) electrodes. 

GER mechanism is a multi-step electron transferring process requiring a lot of active sites as well as abundant electroactive surface area. Additionally, as we deduced from the GER mechanism analysis, the presence of metal oxidative species is essential for its promotion. Thus, the sensing properties (sensitivity, detection limit, etc.) totally depend on the route of the mass and charge transport between M(II)/M(III) or M(III)/M(IV). Thus, the key-requirement is this route to be of low internal resistance and at the same time to offer the capacity for as many as possible active sites. So, a porous structure and a great number of functional groups, can allow higher mass and charge transfer. 

In Table 1 the electrocatalysts that exhibited the highest sensitivity values are reported. The electrocatalysts were classified following a descending sensitivity row. Moreover, their respective limit of detection, the applied potential at which the glucose sensing explored, and the synthesis method are also written (giving the respective number of sub-section). 

It is characteristic that the highest sensitivity values were exhibited by direct growth of graphene onto metallic substrates. This synthesis method has the great advantage that the electrode can be used directly for the detection of glucose without being necessary a transfer process of it, as it is required for many other electrocatalysts. Avoiding this extra transfer step, a possible destruction of graphene (or its derivatives) lattice as well as its contamination, are evaded. Another important advantage is the lack of any binder during the synthesis procedure. Thus, the internal resistance of the electrode can be significantly lower compared to the other methods and consequently glucose sensor’s sensitivity can be extremely enhanced. However, the metal impurities that may remain from the substrate constitutes one of the main challenges that should be overcome. The use of alternative substrates was suggested by a few research groups. Thus, according to our research, it can be stated that Ni foam is a promising substrate, that it is expected to be extensively examined in the future. 

In the pursuit of increasing the available specific surface area some research groups suggested the in-situ growth of an element into graphene sheets. The main element that is currently used in this synthesis strategy is the MOFs. Despite that MOFs have large surface area, high porosity and other chemical and physical properties that make them attractive for the electrochemical sensors’ development; at the same time, they present instability, and they cannot be easily controlled in order specific structures, such as hierarchical, to be obtained. This is a great challenge that should be overcome to explore all their possibilities that their special structure offers. The functionalization of them seems to enhance in-situ MOF/graphene electrocatalyst’s activity. While there is a new trend to in-situ fabricate carbon instead of MOF, which also increases the graphene nanosheets specific surface area. 

On the other hand, laser-induced graphene allows the on-site construction of electrodes onto flexible substrates/platforms. Additionally, the raw materials are of lower cost without requiring any post-treatment steps and it seems to gain significant ground as a synthesis strategy. In case that graphene functionalization is caused by laser tools, the process is accurately controllable, costs much less than other methods using chemical agents. Additionally, this method offers also great reproducibility. However, the technique of metallic nanoparticles addition to LIG structure is still under investigation. One of the main challenges that should be overcome is the anisotropic conductivity of the LIG 3D structure, which dependent on the preparation method that is followed, could affect the preparation time and the uniformity of the metallic nanoparticles or layer onto graphene surface. It should be mentioned that the high price of the laser equipment prohibits this method to be extensively investigated, but we expect much more works to be released.

Since the internal resistance should be as much as lower can be, the combination of conducting polymers with graphene is a trustworthy synthesis method. Some of the conducting polymers have chemical stability along with tunable conductivity and are of low cost, as well. However, the kind of polymer material is an important key parameter that strongly affects a glucose sensor performance. In the future more works seem to explore various polymers and their role to the graphene material for increasing glucose electrodes sensing abilities. According to this method rGO is more favourable than graphene, as displays higher dispersing ability to polymers, that leads to better results.

## 4. Conclusions

Graphene (or derivatives) structure offer conduction channels improving GER kinetics. More precisely graphene (or its derivatives) significantly reduces electrode’s internal resistance enabling GER via the formation of redox pair peaks, corresponding to the M(II)/M(III) or (M(III)/M(IV)) couple formation. As the resistance is low the charge transfer becomes easier and the metal oxide couples that are responsible for GER progress, are created faster. These faster processes lead to higher stability, sensitivity as well as quicker response of glucose electrochemical sensor operation. For faster and continuous metal couples formation and so higher sensitivity four synthesis methods are mainly recognized among literature works: (i) direct growth of graphene (or oxides) on metallic substrates, (ii) in-situ growth of metallic nanoparticles into graphene (or oxides) matrix, (iii) laser-induced graphene electrodes and (iv) polymer functionalized graphene (or oxides) electrodes. Comparatively, the direct growth of graphene onto metallic substrates and the laser induced graphene electrodes synthesis methods, are more mature. Those two methods use fewer chemical reagents and present higher reproducibility. Moreover, the electrode that presented the highest sensitivity to glucose detection was prepared via the direct growth of graphene onto metallic substrate. 

## Figures and Tables

**Table 1:** Electrocatalysts classification with descending sensitivity and synthesis method according to the current review.

Electrocatalyst	Sensitivity (μAmM^−1^cm^−2^)	LOD (μM)	Potential (V vs. Ag/AgCl)	Synthesis Method	Ref.
(Ag⊥@NFG)S	2 × 10^11^	0.002	0.050	2.1	[54]
CuFe-O/Gr/NF hybrid	368,000	0.008	0.650	2.1	[41]
NiNPs/ERGO	185,200	0.040	0.550	2.1	[32]
Ni_3_S_2_/IL-graphene	25,343	0.161	0.370	2.4	[93]
Ni_2_P/G	7234	0.440	0.300	2.2	[61]
Cu/Ni-EG/pNi	6161	0.460	0.550	2.1	[53]
PDDA-graphene/CuO	4982.2	0.200	0.580	2.4	[87]
DLEG-CuNCs	4532.2	0.250	0.550	2.3	[82]
NiO-NC-rGO	4254	0.071	0.500	2.4	[35]
Cu^+2^/PANI/rGO/FR4	4168.37	4.930	0.660	2.4	[95]
Co(OH)_2_/rGO film	3354	1.000	0.500	2.2	[22]
CoO-Co-NC-rGO	3172	0.340	0.600	2.2	[96]
Ag–PANI/rGO	2766.4	0.790	0.500	2.3	[89]
ZIF67@GIS	2718.3	0.086	0.600	2.2	[70]
rGO@Co_3_O_4_-NC	2563	0.0504	0.650	2.2	[17]
CuCo_2_O_4_/PrGO-10	2426	0.150	0.592	2.2	[29]
Ni(OH)_2_/3DGF	2366	0.320	0.454	2.2	[65]
GS/GNR/Ni	2300	0.0025	0.500	2.2	[71]
Ni/NiO-rGO	1997	1.800	0.550	2.2	[31]
GS@ZIF-67	1521.1	0.360	0.592	2.2	[63]
LSG/Cu-NPs	1518	0.350	0.642	2.3	[97]
Co/3D Gr	1411.2	2.700	0.600	2.3	[98]
Ni/C/rGO	1211.41	0.340	0.592	2.2	[34]
CuO_0.1_/LSG	764.331	0.100	0.400	2.3	[99]
PEDOT-ERGO	696.90	0.120	−0.200	2.4	[100]
Cu NPs-LIG	495.0	0.390	0.500	2.3	[39]

## Data Availability

No new data were created or analyzed in this study. Data sharing is not applicable to this article.
